# Long-Term Follow-Up of Flexible Bronchoscopic Treatment for Bronchial Carcinoids with Curative Intent

**DOI:** 10.1155/2009/782961

**Published:** 2010-02-07

**Authors:** Leonardo Fuks, Oren Fruchter, Anat Amital, Benjamin D. Fox, Nader Abdel Rahman, Mordechai R. Kramer

**Affiliations:** The Pulmonary Institute, Rabin Medical Center, Beilinson Campus, Petah Tiqwa 34987, Israel

## Abstract

*Background*. Typical pulmonary carcinoids represent less than 5% of primary lung tumors. In patients with typical bronchial carcinoid, formal surgical resection still remains the gold-standard treatment. Data regarding long-term outcome in using flexible bronchoscope-based modalities under conscious sedation is very limited. *Objectives*. We sought to investigate, over extended follow-up period, the effectiveness of endobronchial resection for carcinoid tumors with curative intent using flexible bronchoscopy. *Methods*. Nd:YAG laser photoresection using flexible bronchoscope under conscious sedation. Follow-up included repeat bronchoscopy every 6 months and chest CT every year. *Results*. Ten patients aged 24 to 70 years with endobronchial carcinoid were treated. The tumor location was variable: 2 left Main bronchus, 1 left upper lobe bronchus, 2 right main bronchus, 2 right middle lobe bronchus and 3 right lower lobe bronchus. No major complications were observed. The patients required between 2 and 4 procedures. Patients were followed for a median period of 29 months with no evidence of tumor recurrence. *Conclusions*. Endobronchial laser photoresection of typical bronchial carcinoids using flexible bronchsocopy under conscious sedation is an effective treatment modality for a subgroup of patients that provides excellent long-term results that are similar to outcome obtained by more invasive procedures.

## 1. Introduction

Bronchial carcinoids account for less than 5% of all primary lung tumors in adults [1]. Roughly 20% of all carcinoid tumors present as purely intralumonal polyp-like bronchial lesions without gross roentgenologically detectable involvement of the bronchial wall and lung [1, 2].

The differentiation of bronchial carcinoid as “typical” or “Atypical” has a clinical and therapeutic importance [3]. Typical carcinoids have an excellent prognosis with a 10-year survival rate of more than 90%. In contrast, atypical carcinoids tend to be more aggressive and metastatic with a 10-year survival rate of less than 60% [4, 5]. 

Although less aggressive than other bronchogenic tumors, atypical carcinoids must be treated as malignant tumors [4]. Surgical resection is the treatment of choice for bronchial carcinoid; however, in the setting of typical and localized endobronchial carcinoid, endobronchial bronchoscopic resection may be considered as an effective and safe alternative to surgery, but data regarding long-term follow-up is scant.

We analyzed our single center experience in the last ten years and retrospectively evaluated the clinical and bronchoscopic outcome of all patients who underwent endobronchial resection of carcinoid tumors with curative intent using flexible bronchoscopy under conscious sedation.

## 2. Materials and Methods

We retrospectively analyzed the medical records of all patients who underwent bronchial resection of typical endobronchial carcinoid at our institute from January 2001 until January 2009. Institutional review board approval was obtained, but specific informed consent was not required for this retrospective study. Informed consent for each bronchoscopy was obtained prior to the procedure.

Selection criteria included the following: proven typical carcinoid without additional pathology confirmed by endobronchial biopsies, strictly endoluminal disease readily accessible to curative bronchoscopic therapy, absence of lymph node enlargement based upon standard CT criteria (<1-cm short axis). 

Carcinoid tumors were defined as “strictly endoluminal” type based upon both CT data (if the tumor was strictly endoluminal without thickening of the bronchial wall and absence of nodal enlargement) and on the endobronchial view [6]. All carcinoids were described as being intraluminal, sessile, or pdunculated by the bronchoscopist. The endobronchial treatment included photoresection using Nd-YAG-laser (30–40 W) by flexible bronchoscopy under conscious sedation (using midazolam 0.04–0.06 mg/Kg and alfentanil 0.5–1.0 mg). Following resection, the base of the tumor was treated by low power setting (15–20 W) to prevent recurrence. If significant bleeding occurred during resection a diluted topical epinephrine (1 : 20000) application was used. A rigid bronchoscope was available on the bronchoscopy suite along with additional safety measures to isolate the bleeding site such as a balloon tamponade catheter. All procedures were done on an ambulatory day-care setting.

All patients underwent repeated bronchoscopic examination at 3, 6, and 12 months after the first session. Biopsies were taken upon each follow-up examination to ensure that no recurrence had occures. If the tumor persisted after four bronchoscopic treatment sessions, surgery was recommended. Biopsies were always taken and one year after the first procedure, patients were referred for follow-up chest CT.

After the first year, we repeated bronchoscopy every 6 months for one more year and then once a year.

## 3. Results

**Figure 1:**
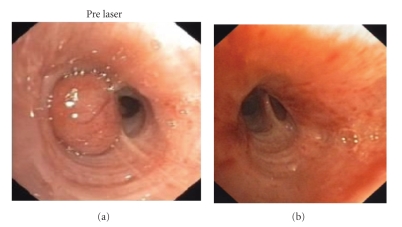
Typical carcinoid obstructing the medial segment of the right middle lobe bronchus of a 52-year-old male (patient no. 6). Before (a) and three months (b) following bronchoscopic laser photoresection by flexible bronchoscopy.

**Figure 2:**
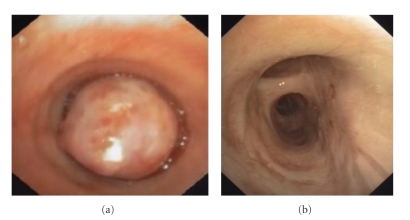
Typical carcinoid in the right main stem bronchus of a 24-year-old female (patient no. 4). Before (a) and six months (b) following 4 sessions of bronchoscopic laser photoresection by flexible bronchoscopy.

We treated 10 patients (3 female and 7 males aged 24 to 70 years old) diagnosed with typical endobronchial carcinoid who were diagnosed as eligible for complete bronchoscopic resection by laser therapy. There were also 3 patients referred for partial resection prior to surgery that were not included in the analysis (Table 1). Symptoms included cough, hemoptysis, and recurrent pneumonia. The tumor was located at various sites (2 Left Main bronchus, 1 left upper lobe bronchus, 2 right main bronchus, 2 right middle lobe bronchus, and 3 right lower lobe bronchus). The patients required between 2 and 4 procedures to achieve complete eradication of the tumor that was observed in all patients. No patient who had been referred for bronchoscopic treatment required surgery. We followed the patients for a median of 29 months (range from 12 to 156 months). The follow-up counting starts upon the first bronchoscopic examination. We plan to follow up all the patients for a total of 5 years according to this protocol, since cure is considered to occur following five years. At the end of the observation period all patients were free of disease both endoscopically and by radiographic criteria. No major complications (such as death, need for mechanical ventilation, or major bleeding) occurred, and no postlaser stenosis was observed. Representative figures of patients no. 4 and no. 6 are given on Figures 1 and 2.

## 4. Discussion

Bronchial carcinoids are rare malignant neoplasms, accounting for 2%–5% of all lung tumors, with an approximate annual incidence of 2.3–2.8 cases per million of the population [1, 2]. The classification in use (WHO 1999) makes a distinction between typical and atypical carcinoids. The Travis-WHO classification [3] is as follows: a typical carcinoid is based upon four elements: a morphological base, a number of mitoses <2 per 10 high power fields (HPFs), absence of necrosis, dimension ≤0.5 cm; an atypical carcinoid has the following morphological characteristics: carcinoid morphology with a number of mitoses ≥2 and <10 per 10 HPFs, and areas of coagulative necrosis. In 10%–15% of cases the tumor can present with regional lymphonodal metastases, and that is why they may be classified as malignant neoplasms, even if with a low degree [3, 4]. 

Distant metastases occur in 15% of cases and are typically located in the liver, bone, adrenal gland, and brain . At present, surgery is the gold standard for treatment of this tumor, with a different approach between typical carcinoids, in which a parenchyma-sparing resection is preferred, and atypical carcinoids, in which a limited resection should be obviated [5]. The concept of bronchoscopic treatment with curative intent for typical bronchial carcinoid has previously reported [6–10]. The aim of this retrospective case study was to assess the safety and efficacy of flexible bronchoscopic ablative therapy for typical endolumonal bronchial carcinoid. 

All studies to date reported high-success rates with good long-term outcome. A summery of previous reports is given on Table 2. We were able to demonstrate that laser photo-resection using flexible bronchoscope is a safe and effective method to treat selected patients with bronchial carcinoids without the need for general anesthesia on an ambulatory service basis. It should be stressed that laser was used to coagulate and vaporize part of the tumor but mechanical resection of the tumor was followed upon the initial procedure to ensure a reliable pathological examination. We showed over a long-term observation period that patients responded well to bronchoscopic treatment and that their survival rates were similar to patients treated with rigid-bronchoscopy ablative techniques [6–10] and conventional surgery [11–13]. Although the use of flexible bronchoscopy increased the number of sessions as compared with the use of rigid bronchoscopy that sometimes enables the physician to complete the procedure in 1-2 sessions.

Strictly, endoluminal typical carcinoids of the lung appear to be quite amenable to bronchoscopic resection. They are often centrally located, with low rates of lymph node invasion and distant metastases [6].

As Cavaliere et al. point out [7], there are two major requirements for curative laser photo-resection for endobronchial carcinoids. The first is that tumor growth is strictly endoluminal as determined by CT, endoscopy, and endobronchial ultrasound. The second is that the base would be small enough and within range of the laser beam. This is best assessed after resection of the endobronchial tumor. If good control of the implantation base is achieved, cure is accomplished consistently. 

Although, from an oncologic point of view, surgery is considered the “gold standard treatment,” bronchoscopic endobronchial resection has been an accepted strategy in bronchial carcinoid treatment [11–13].

Parenchyma-sparing resection is an important goal when dealing with endobronchial tumors and must be the first option whenever feasible [13].

The main limitation of the current study is its retrospective design and the fact that its conclusions advocating flexible-bronchoscopy treatment are based on a single-center experience. 

In contrast to a previous recommendation that flexible bronchoscopy should be used only as an complementary tool for rigid bronchoscopy-based techniques [7], we showed that flexible bronchoscopic therapy under conscious sedation may be considered as a tissue-sparing treatment alternative for rigid bronchoscopy and formal surgery in a selected group of patients who present an intraluminal, typical bronchial carcinoid. Usually, more than a single endoscopic session is required to achieve cure, and patients should be closely and regularly monitored following the procedure.

## Figures and Tables

**Table 1:** Demographic and clinical characteristics of study group patients.

No.	Age/gender	Location	Sessions	Follow-up (month)
1	70/f	LUL	4	72
2	32/f	RLL	2	24
3	30/m	RLL	2	12
4	24/f	RMSB	4	24
5	35/m	RLL	2	12
6	52/m	RML	3	12
7	37/m	LMSB	4	108
8	36/m	LMSB	3	24
9	28/m	RML	2	24
10	42/m	RMSB	3	20

LUL: left upper lobe; RLL: right lower lobe; RMSB: right main stem.

**Table 2:** Summery of studies regarding curative bronchoscopic therapy for bronchial carcinoids.

Studies	No. of patients	Median follow-up (months)	Long-term success rate	Technique
Sutedja et al. [6]	11	70	55%	RB+laser or mechanical under GA
Cavaliere et al. [7]	38	24	92%	RB+ laser under GA
Van Boxem et al. [8]	19	29	73%	RB+ laser under GA
Luckraz et al. [9]	28	105	94%	RB+MR under GA
Bertoletti et al. [10]	18	55	96%	RB+ cryotherapy under GA (14 patients)
				FB+ cryotherapy (4 patients)
Current study	10	24	100%	FB+ laser under CS

RB: rigid bronchoscopy; GA: general anesthesia; MR: mechanical resection; FB: flexible bronchoscopy; CS: conscious sedation.
